# Infectious eye disease in the 21st century—an overview

**DOI:** 10.1038/s41433-024-02966-w

**Published:** 2024-02-14

**Authors:** Gerry Clare, John H. Kempen, Carlos Pavésio

**Affiliations:** 1 Moorfields Eye Hospital, London, UK; 2 Department of Ophthalmology and Schepens Eye Research Institute, Massachusetts Eye and Ear Infirmary; and Department of Ophthalmology, Harvard Medical School, Boston, MA USA; 3 Sight for Souls, Bellevue, WA USA; 4 MCM Eye Unit; MyungSung Christian Medical Center (MCM) Comprehensive Specialized Hospital and MyungSung Medical College, Addis Ababa, Ethiopia; 5 Department of Ophthalmology, Addis Ababa University School of Medicine, Addis Ababa, Ethiopia

**Keywords:** Infectious diseases, Eye diseases

## Abstract

Infectious diseases affecting the eye often cause unilateral or asymmetric visual loss in children and people of working age. This group of conditions includes viral, bacterial, fungal and parasitic diseases, both common and rare presentations which, in aggregate, may account for a significant portion of the global visual burden. Diagnosis is frequently challenging even in specialist centres, and many disease presentations are highly regional. In an age of globalisation, an understanding of the various modes of transmission and the geographic distribution of infections can be instructive to clinicians. The impact of eye infections on global disability is currently not sufficiently captured in global prevalence studies on visual impairment and blindness, which focus on bilateral disease in the over-50s. Moreover, in many cases it is hard to differentiate between infectious and immune-mediated diseases. Since infectious eye diseases can be preventable and frequently affect younger people, we argue that in future prevalence studies they should be considered as a separate category, including estimates of disability-adjusted life years (DALY) as a measure of overall disease burden. Numbers of ocular infections are uniquely affected by outbreaks as well as endemic transmission, and their control frequently relies on collaborative partnerships that go well beyond the remit of ophthalmology, encompassing domains as various as vaccination, antibiotic development, individual healthcare, vector control, mass drug administration, food supplementation, environmental and food hygiene, epidemiological mapping, and many more. Moreover, the anticipated impacts of global warming, conflict, food poverty, urbanisation and environmental degradation are likely to magnify their importance. While remote telemedicine can be a useful aide in the diagnosis of these conditions in resource-poor areas, enhanced global reporting networks and artificial intelligence systems may ultimately be required for disease surveillance and monitoring.

## Abstract (zh)

眼部传染病通常会导致儿童和工作年龄人群的单侧或不对称视力丧失。这类疾病包括病毒性、细菌性、真菌性和寄生虫性疾病, 既有常见病, 也有罕见病, 总体来说, 这些疾病占全球视觉负担的一大部分。即使是在眼科专科诊疗中心, 诊断也常具挑战性, 而且许多疾病的表现具有高度区域性。在全球化时代, 了解各种疾病的传播方式和感染的地理分布对临床医生具有指导意义。目前, 在全球视力障碍和失明发病率的研究中, 眼部感染性疾病的影响未能得到很好的体现, 可能这些研究侧重于50 岁以上人群的双眼性疾病。此外, 在许多情况下很难区分传染性疾病和免疫性疾病的区别。由于传染性眼病可预防, 且常影响年轻人, 我们认为在未来的流行病学研究中, 应将其视为一个独立的类别, 包括将预估残疾调整寿命年数 (DALY) 作为衡量总体疾病负担的标准。眼部感染的例数受到疾病爆发和地方性传播的独特影响, 其控制往往依赖于相关的领域, 这远远超出眼科范围, 包括疫苗接种、抗生素发展、个人医保、病媒控制、大规模药物管控、食品供应, 环境和食品卫生、流行病学地图等多个领域。此外, 全球变暖、冲突、粮食匮乏、城市化和环境退化等预期影响可能会增加其重要性。虽然远程医疗可以在资源匮乏地区在这些疾病的诊断方面提供有效辅助, 但疾病监测最终可能需要强化的全球报告网络和人工智能系统来完成。

## Introduction

The eye is susceptible to infections caused by a bewildering spectrum of organisms, from prions [1] to arthropods [2] (Table 1). Pathology may be restricted to the ocular tissues, or manifest in the eye as part of a systemic infectious disease, with impact ranging from minor nuisance to sight impairment or death [3]. While any structure along the visual pathways may be the focus of an infection, these disease presentations principally fall into one of three distinct anatomical categories: external eye, intraocular structures including the optic nerve, and ocular adnexae, although all three may be involved simultaneously. Many of these infections occur universally, whereas others occur only within certain geographic parameters such as the tropical belt, and seldom surface in temperate countries. A few, like cysticercosis and soil-transmitted helminthiases, are categorised as neglected tropical diseases [4].

**Table 1:** Examples of ocular infections causing sight loss.

Classification	Diagnostic category	Organisms involved
Viruses	Keratitis	Measles virus, herpesviruses, adenovirus
	Cicatrising conjunctivitis	Adenovirus, herpes simplex
	Uveitis	Herpesviruses, rubella, HIV, Ebola, Chikungunya, Zika
	Retinitis	Herpesviruses, Rift Valley Fever virus, Zika virus, Chikungunya, measles
	Foveolitis	Dengue
	Chorioretinitis	West Nile virus, Ebolavirus
	Orbital apex syndrome, optic neuritis, scleritis	Varicella zoster virus
	Post-infectious maculopathies	Influenza, Coxsackie, SARS-CoV2
	Retinal vascular occlusion	Dengue, SARS-CoV2
	Congenital retinopathy and/or retinal lesions	Rubella, Zika, HSV-2
Bacteria and fungi	Trachoma	*Chlamydia trachomatis*
	Keratitis	Various e.g., *Staphylococcus aureus, Fusarium* spp.
	Endophthalmitis	Various e.g., coagulase-negative staphylococci, *Candida albicans, Klebsiella* spp.
	Orbital cellulitis	Various e.g., *Streptococcus pneumoniae, Haemophilus influenzae*
	Neuroretinitis	Spirochaetes (e.g., tick-borne borrelioses, leptospirosis), *Bartonella* spp., rickettsioses
	Ocular syphilis (placoid chorioretinitis, optic neuritis, multifocal retinitis, vitritis, granulomatous uveitis)	*Treponema pallidum*
	Hypopyon uveitis	Leptospirosis (Weil’s disease)
	Multifocal retinitis	Rickettsioses, *Bartonella* spp.
	Ocular tuberculosis (granulomatous uveitis, occlusive retinal vasculitis, serpiginous-like and ampiginous choroiditis, choroidal granulomas, and/or optic nerve granuloma)	*Mycobacterium tuberculosis*
	Ocular leprosy, erythema nodosum leprosum, iris leproma	*Mycobacterium leprae*
	Choroidal granuloma	*Brucella* spp., TB
	Multifocal choroiditis	*Nocardia* spp., paracoccidioidomycosis, coccidioidomycosis, presumed ocular histoplasmosis syndrome
	Post-streptococcal uveitis	β-haemolytic (Group A) streptococci
	Optic disc oedema	*Tropheryma whipplei*
**Parasites and arthropods**	Onchocerciasis	*Onchocerca volvulus*
	Ocular toxoplasmosis (chorioretinitis)	*Toxoplasma gondii*
	Keratitis	*Acanthamoeba* spp., *microsporidia* spp.
	Post-kala azar ocular leishmaniasis	*Leishmania donovani, Leishmania infantum*
	Ocular toxocariasis	*Toxocara* spp.
	Diffuse subacute neuroretinitis	Various, e.g., *Toxocara* spp., *Baylisascaris procyonis*, soil-transmitted helminths, non-human hookworms
	Orbital and ocular tapeworm infections	Cysticercosis (*Taenia solium*), *Echinococcus granulosus*, *Spirometra* spp.
	Presumed trematode-induced granuloma	*Schistosoma* spp., *Procerovum varium*
	Orbital and ocular roundworm infections	*Trichinella* spp., *Dirofilaria* spp., *Angiostrongylus* spp., *Gnathostoma* spp.
	Ocular pentastomiasis	*Armillifer armillatus*, *Linguatula serrata*
	Ophthalmomyiasis	Various, e.g., sheep bot fly *Oestrus ovis*

As global warming progresses, however, geographic boundaries may shift [5]. In addition, widespread international travel and migration makes geographic boundaries porous, with the result that clinical presentations more commonly associated with tropical climes can present and must be recognised worldwide [6]. Infectious diseases that can manifest in the eye such as tuberculosis (TB) and measles tend to occur disproportionately among persons from developing countries with incomplete healthcare coverage and among migrants from these countries [7]. Moreover, emerging infectious diseases (EID) including arthropod-borne viral (arboviral) infections (e.g., Zika virus) and diseases of presumed zoonotic origin (e.g., Ebolavirus) have given rise to unexpected ocular pathologies [8–10]. While recognition thereof can be life-saving or life-changing, a failure to understand its implications can lead to inappropriate immunosuppression, with devastating consequences. It therefore behoves ophthalmologists everywhere to have a working knowledge of the vast spectrum of infectious diseases that can affect the eye.

Correct diagnosis is the critical first step in directing patients towards appropriate healthcare services for sight- or life-preserving treatment, reducing transmission, or even reporting an outbreak [11]. Once an infectious entity is suspected, a specific history guided by clinical and epidemiological risk factors should be elicited to determine possible relevant routes of potential exposure, and appropriate investigations requested to aid consultation and further care. Specialist investigations and multidisciplinary management may be necessary but are often not possible in low-and middle-income countries, which bear the brunt of global visual impairment [12]. Whereas ophthalmologists in rich countries benefit from a plethora of aids to diagnose infectious disease, such as DNA analysis of ocular samples and CT-PET scans to identify avid lymph nodes, medical staff in resource-poor settings may be guided only by clinical acumen and epidemiological knowledge [13]. The challenges are substantial, and although they are being addressed through innovative public health initiatives, much work remains to be done at a grassroots level [14].

From an epidemiological perspective, infectious diseases trail far behind the main global causes of avoidable moderate-to-severe distance visual impairment (MSVI) and avoidable blindness, estimated in a large meta-analysis to affect 553 million and 43 million people, respectively in 2020 [15]. Due to lack of sufficient data, the meta-analysis is based on population-based surveys of eye disease in people aged 50 and above, and the definition of sight impairment stipulates a visual acuity threshold for the better-seeing eye, thus neglecting the burden of conditions affecting only one eye [16]. Important infectious entities causing bilateral visual impairment remain trachoma and onchocerciasis, yet both are in sharp decline and anticipated to be under control by 2030 [12]. Moreover, both diseases are located in geographical pockets of high risk, in contrast to infectious eye diseases with a more global distribution, such as herpes and syphilis. While this underscores a shift in visual burden towards non-communicable diseases, the true global visual burden of infectious diseases is most likely underestimated for several possible reasons. Most significantly, infectious eye conditions are often unilateral, and are therefore not captured in prevalence studies using commonly adopted definitions of sight impairment. Monocular visual impairment is now officially recognised as a disability by WHO [17], and is much more common than bilateral visual impairment [18]. Moreover, in contrast to sight-threatening conditions acquired in maturity, many infectious eye diseases are likely to be evenly distributed across all age groups, and epidemiological or population-based studies focusing on the over-50s may underestimate both their statistical significance and economic impact. For example, studies from low vision services in Latin America indicate toxoplasmosis as one of the leading causes of childhood blindness, yet this valid source of information is not currently incorporated into global estimates. Since toxoplasmosis is common and globally distributed, its toll on vision may be significantly underestimated (especially in terms of years of vision loss), possibly even vying with trachoma and onchocerciasis as a leading infectious cause of sight impairment and blindness worldwide. Determining the true global visual burden of ocular toxoplasmosis will be a sizeable challenge.

There are several other reasons for this suggested attribution bias as the following examples demonstrate. In many parts of the world where ocular infections are common, ophthalmic services in aggregate are often underdeveloped or absent altogether, and diagnoses may easily be missed or simply unreported [13]. Ocular infections can cause secondary pathologies, such as cataract, glaucoma, or retinal detachment, to which sight loss might be attributed without recognition of the underlying cause [19]. Common medications used to treat ocular infections, such as ethambutol and linezolid, may themselves cause visual loss [20]. Para- or post-infectious syndromes may not be identified and assumed to be auto-immune non-infectious presentations. The role of immune-mediated pathology following infections is complex and incompletely understood [21]. The global visual burden of EIDs such as dengue fever, Ebolavirus disease and coronavirus disease remains poorly understood [8, 22, 23]. Estimating the global visual burden of infectious disease is, therefore, likely to be an extremely complex undertaking.

Although data are currently sparse, it is likely that if one were to combine in one category all unilateral and bilateral visual loss due to infectious causes of keratitis [24], optic neuritis [25], uveitis [26] and orbital cellulitis [27], infectious disease would feature more prominently as a cause of the global visual burden. Progress has been achieved across multiple domains in reducing the burden of trachoma, cytomegalovirus retinitis, and onchocerciasis [13], suggesting that sight impairment from other infectious causes should be similarly preventable by collaboration between various disciplines. In this respect, infectious causes of eye disease stand out from other sight-threatening conditions, and in future prevalence studies, it would therefore be useful to consider them in aggregate.

## Viral ophthalmic diseases

Many viruses cause ophthalmic disease, including adenovirus, influenza A, SARS-CoV-2, herpesviruses, HIV, measles, arthropod-borne viruses (arboviruses), zoonotic viruses, and others [23, 28]. The herpesviruses and adenoviruses possess double-stranded DNA, whereas the bulk of the remainder of viruses affecting the eye (flaviviruses, influenza, measles, filoviruses, and others) are enveloped single-stranded RNA viruses [29]. Certain RNA viruses, suggested to be inherently more mutation-prone, have been implicated in EIDs and may prove to be causative pathogens of future pandemics [8, 23, 30].

While adenovirus, influenza, and coronavirus infections typically pass without lingering effects, they occasionally have sight-threatening sequelae such as corneal scarring [31], acute posterior multifocal placoid pigment epitheliopathy [32], and retinal vascular occlusion [33], respectively. These complications may be rare, but as infections with these viruses are common, their visual burden around the world may be significant. While vaccination might be expected to mitigate visual loss from these and other viruses, vaccines themselves can precipitate ocular complications leading to visual loss [34].

The herpesviruses cause a range of pathologies, from self-limiting dendritic corneal ulcers to bilateral acute retinal necrosis (ARN), leading to visual impairment, and even complete blindness globally [35–38]. This group includes varicella zoster and herpes simplex type 1, the most common causative agents of ARN in adults, and herpes simplex type 2, an important cause of ARN in children following neonatal exposure [39]. These are common pathogens with a worldwide distribution, serological exposure increasing with age [40]. It has been calculated that, in 2016, 230,000 people around the world suffered uniocular visual impairment because of newly diagnosed HSV keratitis [35]. Infection with these viruses can lead to disseminated multisystem disease with florid ocular involvement, in some cases leading to death, especially in the context of immunodeficiency, immunosuppression, or immunosenescence [41]. While there may be a role for recombinant vaccines in preventing ocular complications and visual loss from varicella zoster infections [42], there have been numerous case reports of ARN and other manifestations of herpetic infection following vaccination [22]. Vaccines are under development for HSV-2 infection which is thought to increase the risk of acquiring HIV [43].

In the same family, cytomegalovirus (CMV) has emerged as a common cause of hypertensive anterior uveitis [44], and also causes a characteristic retinitis in immunodeficient individuals, leading to a substantial visual burden especially in poor countries [37]. These infections are treatable with a combination of anti-viral drugs, corticosteroids, and anti-glaucoma medications. Screening and treatment programmes by HIV physicians have been effective in some areas [45]. In addition, congenital CMV infection is associated with ophthalmological disorders including retinochoroiditis and visual impairment in a proportion of cases [46]. While the possible role of Epstein-Barr virus in causing retinal disease is controversial, as it can often be detected in non-infectious uveitis, it is strongly associated with nasopharyngeal cancer and lymphomas that can invade ocular tissues [47, 48]. Similarly, human herpesvirus-8 is associated with Kaposi sarcoma, which occasionally involves the conjunctiva and/or the eyelid [49].

Although HIV causes retinopathy and anterior uveitis in its own right [50], the bulk of ocular damage in HIV disease is from opportunistic infections such as cytomegalovirus, varicella zoster, *Cryptococcus neoformans*, *Pneumocystis jirovecii*, human herpesvirus 8, microsporidia, *Toxoplasma gondii*, and others, as well as infection-induced neoplasia [37, 51–54]. In addition, visual loss may occur as a result of immune recovery inflammatory syndromes following treatment of HIV with modern combination anti-retroviral therapy, including immune recovery uveitis [55] and paradoxical worsening of TB [56]. Visual loss may also occur because of drug-induced uveitis, most commonly with cidofovir or rifabutin [57]. While recent diagnostic and therapeutic developments have allowed many people with HIV to enjoy an apparently normal life expectancy, health inequalities dictate that in several parts of the world, coverage is still incomplete. Consequently, ophthalmic complications of HIV disease persist, producing visual impairment in an unknown proportion of the approximately 40 million people living with HIV worldwide, probably more so in poor countries without screening programmes for CMV retinitis [13]. As the new brands of anti-retroviral medications become more available across the globe, we can expect this proportion to fall.

Another retrovirus, human T lymphotropic virus type 1 is one of the most common causes of retinal vasculitis and vitritis in endemic areas such as Japan, frequently leading to visual loss [58].

Worldwide, the measles virus has been one of the most common causes of blindness in childhood in at-risk populations [59]. This occurs because infection can cause a precipitous loss of vitamin A to which children with already low reserves are especially vulnerable [60]. The acute vitamin A deficiency leads to a progressive spectrum of ocular pathology, termed xerophthalmia, from night blindness and severe ocular surface dryness (xerosis) to corneal ulceration, keratomalacia, and corneal scarring, along with permanent retinal structural changes. Cell-mediated immunity also is compromised, making the cornea susceptible to secondary infection and necrosis. In addition to exacerbating hypovitaminosis A, the measles virus can directly cause keratitis, retinitis and optic neuritis. The mainstay of prevention in malnourished populations is measles vaccination, together with vitamin A supplementation given at the time of vaccination, as well as in fortified food. While vitamin A deficiency worldwide has been decreasing worldwide [61], the impact of food poverty due to conflict and global warming may be significant this century. In addition, measles vaccination rates have been hit by misinformation campaigns, with unclear consequences for future measles epidemics [62].

Despite the development of an effective vaccine against the rubella virus over half a century ago, congenital rubella syndrome persists as a disease with devastating ocular consequences [63] in a few countries where vaccine uptake remains low [64]. Pregnant women infected with rubella in the first trimester are most at risk of transmitting the virus to the foetus, resulting in congenital ocular manifestations such as cataract, microphthalmos and pigmentary retinopathy [65]. More recently, an association has emerged between rubella and Fuchs uveitis syndrome, evidenced by the finding of an excess of rubella antibodies compared to serum levels, as well as the rubella genome, in the aqueous humour of eyes with clinically defined Fuchs uveitis syndrome [66]. A decrease in this syndrome has been reported in the United States following the introduction of the rubella vaccination programme in 1969 [67].

Dengue is the most common arbovirus worldwide, infecting up to 400 million people every year following the bite of *Aedes* spp. mosquitoes [68], mainly in urban centres in the tropical belt. These vectors proliferate during wet seasons, driving dengue epidemics. The number of cases of dengue has been increasing year after year, principally due to global warming favouring the expansion of the habitat of its mosquito vectors [69]. Typically, primary infection with one of the four viral serotypes is mild. A second infection with a different serotype, however, can produce a more severe clinical picture including haemorrhage and death, which has been attributed to antibody-dependent enhancement [70]. This mechanism is a potential obstacle to the development of a dengue vaccine. A small proportion of infected persons will have some degree of ocular involvement, from a self-limiting multifocal retinitis and vitritis to a posterior pole ischaemic retinal vasculitis and foveolitis, with outcomes ranging from a transient disturbance to permanent central blindness [71, 72]. In one outbreak of dengue serotype 1, the prevalence of maculopathy was reported to be 10% [73]. The risk factors leading to ocular complications are unknown [74], but it seems plausible that severe ocular involvement may be more likely following secondary infection. As the development of an effective vaccine has so far proven elusive [75], efforts at containing dengue have focused on limiting the ability of the mosquito vector to transmit the virus, for example by infecting mosquitoes with a ubiquitous endosymbiotic bacterium, *Wolbachia* spp. [76].

Another of the viruses transmitted by *Aedes* mosquitoes, the chikungunya virus has a similar environmental suitability map to dengue [77] but differs from dengue in that it has an additional sylvatic life cycle involving non-human primates. Chikungunya infection is typically self-limiting but can be associated with ocular findings causing visual loss, such as intraocular inflammation, multifocal retinitis and optic neuritis [78]. Far less common than dengue, the closely related Zika virus is also transmitted by *Aedes* spp., and its sight-threatening complications include uveitis and congenital malformations of the eye [79]. Zika virus infection causes anterior uveitis in acquired disease, and macular lesions in congenital disease. In contrast, West Nile virus is transmitted to humans from infected birds by *Culex* spp. mosquitoes. Infection produces a characteristically linear chorioretinitis [80]. In each case, the expansion of the mosquito vector habitat will determine the geographic spread of the infection, while climatic events such as precipitous rainfall and flooding may be a significant factor in driving future vector-borne epidemics [81].

Outbreaks of Rift Valley fever typically occur in Africa and the Middle East during periods of high rainfall leading to a massive increase in the proliferation of *Aedes* and *Culex* vectors, which then transmit the virus from livestock to man in high numbers [82]. In a small proportion of cases (estimated at around 1 to 2%), infection with RVF virus causes a characteristic macular retinitis, which is frequently bilateral and can lead to central blindness [83].

Recent outbreaks of presumed zoonotic viruses that predominantly spread directly from person to person, such as Ebolavirus (thought to be initially transmitted to humans by fruit bats) [9] and SARS-CoV-2 (zoonotic origins disputed) [10], have also been associated with ocular morbidity. In the case of Ebola, the filovirus can survive in the eye for several months after infection (similar to Marburg), eliciting a spectrum of signs from mild chorioretinal scarring to severe panuveitis in a high percentage of survivors, many of whom had secondary complications such as cataract, glaucoma and retinal detachment [84, 85].

Infection with SARS-CoV-2, on the other hand, may precipitate a hypercoagulable state indirectly resulting in retinal arterial and venous occlusions, as well capillary ischaemia possibly linked to paracentral acute middle maculopathy and acute macular neuroretinopathy [33]. Vaccination is now a key strategy to limit the morbidity of these viruses, although vaccines themselves have been reported to be associated with ocular pathologies similar to those found following SARS-CoV-2 infection [33, 86]. Proving causality, however, remains challenging.

Numerous other viruses have been reported to cause ocular pathology and visual loss, such as Coxsackie virus [87], a putative cause of unilateral acute idiopathic maculopathy. The role of viral illness in presumed post-infectious ophthalmological entities such as multiple evanescent white dot syndrome remains incompletely understood [88, 89]. While many patients recover vision spontaneously following post-infectious syndromes, a minority of patients lose vision permanently.

## Bacteria and fungi

The global visual burden of trachoma, caused by repeated conjunctival infections with serological variants of the obligate intracellular bacterium *Chlamydia trachomatis* has been well documented [90, 91]. The *Musca sorbens* fly, which feeds on human mucosal secretions and preferentially lays its eggs in human faeces, is thought to act as a mechanical vector for the bacterium in endemic areas, especially in areas where sanitation is poor and open defecation is practiced [92]. Repeated infections (around 150 to 200) cause progressive tarsal conjunctival scarring, leading to entropion and trichiasis, which in turn scars the corneas leading to sight loss, typically in middle age. According to WHO, trachoma is currently responsible for the blindness or visual impairment of about 1.9 million people in 42 countries, and about 1.4% of all blindness worldwide at an annual cost of 2.9 to 5.3 billion US dollars. The visual burden, shouldered mostly in sub-Saharan Africa, had been decreasing every year [93] until the worldwide SARS-CoV-2 pandemic of 2020 when progress was briefly disrupted. Key to this progress is the SAFE strategy, a treatment and prevention programme adopted by WHO in 1993. The strategy prioritises Surgical procedures, including epilation and posterior lamellar tarsal rotation for trichiasis and cicatricial entropion, respectively [94], mostly carried out by trained nurses, Antibiotic treatment (e.g., oral azithromycin), awareness of hygiene measures such as Face washing, and Environmental improvements including closed latrines and clean water sources [95]. While it has not been possible to eliminate the disease as a public health problem thus far, the WHO Alliance for the Global Elimination of Trachoma by 2020 now aspires to eliminate it by 2030 [12, 96]. If this is achieved, it will represent the culmination of a vast combined effort by various international, governmental and non-governmental partners to carry out epidemiological surveillance, evaluate projects and mobilise resources, and could provide a template for limiting preventable visual impairment due to other less common infectious causes, for example by promoting reporting of relevant data on infectious eye disease to WHO.

Several sight-threatening ocular pathologies, including infectious keratitis, orbital cellulitis and endophthalmitis are caused by a wide variety of different pathogens, including bacteria, fungi, protists and viruses; co-infection is also possible. Many of the causative pathogens are universal, whereas others exhibit considerable variation in geographic distribution [97]. In the tropics, these conditions may be caused by organisms unfamiliar to ophthalmologists in temperate countries. As an example, *Burkholderia pseudomallei*, a Gram-negative bacillus acquired through direct contact with contaminated soil and water, is a known cause of infectious keratitis, endophthalmitis and orbital cellulitis in Thailand [98], yet its geographical distribution outside southeast Asia and northern Australia remains poorly understood [99].

Infectious keratitis, commonly called corneal ulceration, is one of the leading global causes of unilateral blindness; it has been described as a ‘silent epidemic’ [100]. The visual burden of infectious keratitis in resource-poor settings greatly exceeds that in rich countries, prompting a proposal to designate this condition as a neglected tropical disease [101]. Common pathogenic causes worldwide include coagulase-negative staphylococci, *Streptococcus pneumoniae*, *Pseudomonas aeruginosa*, *Staphylococcus* spp., herpes simplex and zoster, *Fusarium* spp., *Aspergillus* spp., *Microsporidia*, *Candida* spp. and *Acanthamoeba* spp., and rarer causes include non-tubercular mycobacteria and *Nocardia* spp., as well as a plethora of other organisms [24]. Risk factors include hypoxia-inducing contact lens wear, while agricultural and other ocular trauma is a more common predisposing factor in resource-poor countries. In a proportion of cases, it is bilateral, notably in neonatal conjunctivitis where the causative pathogen may be *Neisseria gonorrhoeae*, which can lead to blindness rapidly and may be resistant to antibiotics [102]. More broadly, the prevalence of multidrug-resistant bacterial keratitis may be increasing [103]. The true burden of infectious keratitis is unknown, in part because epidemiological studies lack the data to disaggregate it from other non-infectious causes of non-trachomatous corneal opacity, estimated to cause 3.2% of global blindness [104]. In areas where bacterial, fungal and other pathogenic causes of infectious keratitis are common, it can be difficult to distinguish these clinically. There is no substitute for microbiological diagnosis, including Gram staining, culture and DNA analysis of tissue samples in identifying the causative pathogen to guide treatment [105]. Wherever this facility is lacking, however, empirical treatments with topical antibiotics and anti-fungals are sometimes the only option to treat infectious keratitis, leading to uncertain outcomes. The emergence of multidrug resistance, as well as the harm done by traditional medicines [106], contribute further to the toll of infectious visual loss. The role of antiseptics such as povidone-iodine in treating infectious keratitis has been evaluated, showing promise against Gram-positive organisms [107].

Orbital cellulitis remains a significant cause of visual morbidity as well as mortality worldwide, more frequently affecting children [108]. It commonly results from the spread of infection from the sinuses and periorbital skin, as well as haematogenous spread from distant sites. Infection may be polymicrobial, although streptococcal and staphylococcal species predominate. Aggressive treatment is typically required to prevent complications such as visual loss, including immediate empirical treatment with intravenous antibiotics and early surgery to drain sinuses and abscesses. This level of care is frequently unavailable in resource-poor settings. Vaccination against *Haemophilus influenzae* type B is one measure judged to have reduced rates of orbital cellulitis in children [27]. In poor countries, however, many children remain unvaccinated [109] and at risk of visual loss.

Invasive fungal causes of rhinosinusitis, often caused by *Mucor* and *Aspergillus* species, are a feature of immunodeficiency, uncontrolled diabetes mellitus and corticosteroid treatment, and saw a resurgence during the 2020 SARS-CoV-2 pandemic attributed to treatment of serious respiratory complications with corticosteroids, notably in South Asia [110].

Infectious endophthalmitis remains a serious possible complication of all penetrating eye injuries and intraocular procedures [111], but it may also arise spontaneously, rarely in otherwise completely asymptomatic individuals, as well as those with serious systemic infections, following haematogenous spread of bacterial, fungal and other pathogens [112]. As a matter of convention, viral and parasitic causes of endogenous infection are usually considered separately as uveitic entities [113]. The list of organisms reported to cause endophthalmitis is long, but commensal coagulase-negative *Staphylococcus* spp. were found to predominate (39.4%) in one large series of culture-positive isolates from a single institution in North America, followed by *Streptococcus viridans* (12.1%) and *Staphylococcus aureus* (11.1%) [114]. Gram-negative organisms and fungi accounted for 10.3% and 4.6% of isolates, respectively. More indolent causes of endophthalmitis, typically with a delayed presentation, include *Cutibacterium acnes* [115]. Regional differences in the percentages of different causative pathogens exist, as exemplified by endogenous *Klebsiella pneumoniae* endophthalmitis secondary to pyogenic liver abscess, often reported to be more prevalent in east Asian countries but which is emerging in several countries around the world [116]. In rich countries, postoperative endophthalmitis is well recognised as a serious complication, and is typically managed as a medical emergency with intravitreal and systemic antibiotics and vitrectomy, with mixed results reflecting the virulence of the causative organism and the timing of presentation. In many resource-poor settings, however, the circumstances surrounding presentations of endophthalmitis may be very different, often leading to poor visual outcomes and impacting on patients’ economic potential [117]. As cataract surgical rates increase around the world, and with the emergence of intravitreal therapies, the incidence of endophthalmitis can be expected to rise concomitantly.

Endogenous fungal endophthalmitis is associated with distinct medical risk factors, including diabetes mellitus, immunosuppression, dialysis and intravenous drug use [118]. Lemon juice used to dissolve opiates and stimulants has long been identified as a source of *Candida* spp. in cases of endophthalmitis among intravenous drug users [119].

Mycobacterial diseases, chief among which is tuberculosis, impose a significant burden on vision worldwide, especially in endemic areas but also in some countries with low endemicity [120]. Approximately 10.6 million people around the world fell ill with TB in 2021 [121], yet significant gaps in our knowledge concerning the global visual burden of ocular TB remain. In part, this is because ocular TB is itself often a challenging concept for ophthalmologists and TB physicians alike [122]. The diagnosis can be straightforward, for example when a choroidal granuloma is found in a patient from an endemic area, and a chest X-ray showing characteristic changes together with a positive immunological test corroborate the ocular findings. At the other end of the scale are clinical presentations that suggest but do not establish beyond doubt a diagnosis of ocular TB, such as occlusive retinal vasculitis, ampiginous, serpiginous-like and multifocal choroiditis, as well as chronic granulomatous uveitis [123]. To complicate matters, radiological and immunological tests (e.g., tuberculin skin test or interferon-gamma release assay, which do not distinguish between active and latent TB) may be negative even in culture-confirmed TB [124]. The Collaborative Ocular Tuberculosis Study group has recently devised a consensus-based decision-making tool enabling users to confidently recommend starting ATT [125]. Once treatment is started, the clinical picture may initially deteriorate due to increasing inflammation, a phenomenon known as paradoxical worsening [126]. The emergence of multidrug-resistant strains of TB further complicates progress, although new drug regimens have recently been found to be non-inferior to standard treatments [127]. The global prevalence of visual impairment secondary to ocular TB, therefore, is a complex question likely to tax even the most assiduous epidemiological researcher.

While it is much less common than TB, leprosy (*Mycobacterium leprae*) also can cause visual impairment, mostly due to neurotrophic keratitis and lagophthalmos secondary to cranial nerve inflammation or bacillary invasion, but also due to direct bacillary invasion of intraocular structures [128]. Following systemic treatment for multibacillary (lepromatous) leprosy, it is possible for bacilli to persist inside the eye, eliciting a severe inflammatory response leading to visual impairment and blindness [129]. A biopsy of iris granulomas (leproma) in patients with chronic uveitis treated for leprosy may occasionally demonstrate viable mycobacteria, indicating a need for further treatment rather than immunosuppressive therapy alone [130]. It is unclear whether these organisms can survive in the eye because of treatment failure, suppressed cell-mediated immunity [131] or possibly anterior chamber-associated immune deviation.

Non-tubercular mycobacteria (e.g., *Mycobacterium abscessus*) have been implicated in severe ocular infections, such as necrotising sclerokeratitis resulting in enucleation [132]. Diagnosis may be delayed, even with tissue biopsies sent for histopathological analysis, as the bacilli can be mistaken for *Corynebacteria* or *Nocardia* spp. on microscopy.

Spirochaetes represent another group of bacteria of great ophthalmological importance, including syphilis, leptospirosis and Lyme disease all of which can have a significant impact on the eyes [133]. Less well known are the tick-borne relapsing fevers, endemic in many tropical and temperate regions and also able to cause ocular inflammation [134].

In the past two decades, there has been a resurgence of syphilis in other high-income countries, linked in part to the success of both treatment and pre-exposure prophylaxis against HIV [135]. This can be associated with a reduction in the use of condoms during high-risk sexual activity, resulting in an increase in other sexually transmitted infections [136]. Ocular syphilis, caused by infection with *Treponema pallidum*, can manifest in various forms, including chronic granulomatous or non-granulomatous anterior uveitis, retinitis, optic neuritis and a characteristic placoid chorioretinitis from a few weeks to several months after primary infection [137, 138]. It is assumed that following infection, treponemes can invade the ocular tissues, including the retina which is part of the central nervous system, and ocular syphilis is therefore considered to be a manifestation of neurosyphilis. Signs of secondary syphilis, such as palmar and plantar rashes, may be present, although ocular syphilis may feature at any stage of the disease. The ocular signs may be subtle and can be missed [139], often leading to counterproductive treatment with systemic corticosteroids. In congenital cases, Hutchison’s triad consisting of interstitial keratitis, malformed teeth and eighth nerve deafness may be present. Serological testing is an essential aspect of diagnosis. False positive treponemal serology tests are possible in cases of endemic treponemal infections such as yaws, bejel and pinta, whereas non-specific serological tests may be positive in certain non-syphilitic conditions [140]. Once positive, treponemal serology does not revert, whereas non-specific assays can be used to monitor treatment success, defined as a fourfold decrease in titre (e.g., from 1:1024 to 1:256). Ocular syphilis must be managed in collaboration with colleagues in sexual medicine, following neurosyphilis treatment protocols [137, 138]. While some visual recovery is possible following treatment, visual impairment can be permanent if the diagnosis is delayed, yet the global visual burden remains unknown.

In recent decades, infections with *Leptospira* spp., one of the world’s most common zoonoses [141], have emerged as a major cause of ocular inflammation and visual impairment, predominantly in tropical zones [142]. It may occur months or even years after primary infection following contact with contaminated water during an epidemic, often presenting with non-granulomatous hypopyon uveitis, although a multitude of ophthalmic signs such as retinal vasculitis and vitritis are recognised associations [143]. Visual prognosis is usually good, but secondary complications such as cataract are common [144]. Although leptospirosis is common worldwide, it is rarely considered - let alone tested for - in the Global North, except perhaps in moderately high prevalence areas like Hawaii. Outbreaks are related to sanitation and are therefore difficult to prevent in many parts of the resource-poor world [145].

Tick-borne borrelioses include Lyme disease and tick-borne relapsing fevers [140], both of which cause inflammatory eye diseases [146]. Lyme disease is prevalent in areas where humans encounter deer infected with *B. burgdorferi* in the United States and *B. afzelii* or *B. garinii* in Europe. Lyme disease is a well-recognised cause of intermediate, posterior and panuveitis, including retinal vasculitis, serous retinal detachment and papilloedema, with late manifestations including peripheral ulcerative keratitis. Tick-borne relapsing fevers are caused by locally endemic *Borrelia* spp. in central Asia, East Africa, the Mediterranean and the Americas. They are rarely considered as causes of uveitis outside these endemic areas, and their burden is unknown.

The tick-borne diseases also include rickettsioses such as the spotted fever group [147]. Mediterranean spotted fever (also known as boutonneuse), caused by *Rickettsia conorii* and transmitted by canine ticks, is prevalent in the Mediterranean basin, the Middle East, central and southern Asia, and sub-Saharan Africa. It can present with focal and multifocal inner retinitis, neuroretinitis and uveitis [148]. Typically, a black eschar is present on the skin indicating a tick bite, and there may be a history of a headache, fever and skin rash. It is one of the most commonly imported rickettsioses by returning international travellers [149].

Cat scratch disease, caused by *Bartonella henselae*, is transmitted directly to humans by cats through biting and scratching, as well as by tick and flea vectors [150], at least between cats. It is a well-known cause of multifocal retinal infiltrates, retinal artery occlusion and neuroretinitis, potentially leading to visual loss [151, 152]. The disease is thought to have a worldwide distribution, although its effects on visual loss in the tropics remain unknown.

Another common zoonosis in the tropical belt, Malta fever is caused by *Brucella melitensis* transmitted by unpasteurised dairy products and may be complicated by focal chorioretinitis and optic nerve swelling [153, 154]. Choroidal granulomas may be present, mimicking tuberculosis. In one series of 1551 patients, ocular involvement, most commonly posterior uveitis, was found in 0.7% of patients with acute brucellosis and in 7.9% of patients with chronic brucellosis, in some cases leading to blindness [155]. Prevention depends on quality control of dairy products and vaccination of livestock in endemic countries [156].

Post-streptococcal syndrome uveitis is an immune-mediated response following a group A β-haemolytic streptococcus pharyngeal infection, which may include bilateral sight threatening non-granulomatous panuveitis, mostly affecting children and teenagers in economically challenged communities [157]. Elevated anti-streptolysin O titres confirm a recent invasive streptococcal infection in these patients [158].

Community-acquired fungal infections rarely can spread to the eye, causing severe visual loss, even in immunocompetent individuals. Culprits include coccidioidomycosis [159] and paracoccidioidomycosis [160], inhaled as spores often in an agricultural setting in hyperendemic areas. In contrast, presumed ocular histoplasmosis is thought to be an immunological reaction following exposure to histoplasma in endemic areas [161], and is a significant cause of visual morbidity. Disseminated histoplasmosis and talaromycosis are more likely to features of profound immunodeficiency [162], while sporotrichosis endophthalmitis can occur because of traumatic inoculation [163].

## Parasites and arthropods

Parasitic infections have a particularly strong association with the tropics, reflecting habitats and climates that favour parasite and vector survival as well as social and environmental conditions that permit human infection. The eye can host both protozoan organisms, which include amoebae, and helminthic parasites, and rarely it can become severely damaged by invasion by arthropods [2]. Some, but not all parasites that affect the eyes are important zoonoses [164].

Two protists, namely *Acanthamoeba* spp. and *Toxoplasma gondii*, cause widespread ophthalmic pathology. *Acanthamoeba* spp. are one of a group of ubiquitous free-living amoebae that occasionally cause fatal encephalitis in humans and are well known as a cause of painful infectious keratitis and scleritis associated with exposure to contaminated water and contaminated contact lens equipment [165, 166]. Acanthamoebal sclerokeratitis may be refractory to treatment and persist for several years, and trophozoites may occasionally invade the eye, leading to severe inflammation and blindness. The incidence is said to be increasing due to the global epidemic of myopia, which has resulted in increased contact lens use [167].

*Toxoplasma gondii* is one of the most common parasites in the world to infect humans and is a major cause of retinochoroiditis and visual loss [168]. It is a well-recognised cause of congenital blindness following primary maternal infection and transplacental spread [169]. It may be acquired in life following exposure to sporulated oocysts in cat faeces or contaminated soil and water, or to tissue cysts through ingestion of undercooked meat, blood transfusion and organ transplantation. Transmission is more common in warm, humid climates germane to the sporulation and prolonged survival of oocysts in the soil. Additional risk factors include the presence of oocysts in drinking water [170], and social factors such as acceptability of eating raw meat [171]. Primary infection may lead to a florid retinochoroiditis and vitritis, especially in immunodeficient and immunosenescent individuals [172], although severe systemic manifestations are possible even in immunocompetent travellers [173]. Typically, the inflammation subsides leaving behind retinal scars which become increasingly pigmented over time. Cysts are present adjacent to the scar, and recurrence of inflammation due to tachyzoite proliferation is not uncommon. In some instances, especially in the setting of immunodeficiency, toxoplasma retinochoroiditis can run an aggressive, protracted course leading to severe visual impairment. Disease acquired during pregnancy poses a serious risk to the unborn child and requires management by the obstetrician to prevent vertical transmission [174].

Post-kala azar ocular leishmaniasis is a protozoal parasitic ocular condition that is not widely known among Western ophthalmologists, but which may affect travellers returning from countries where endemic or sporadic visceral leishmaniasis occurs in the population [175]. Visceral leishmaniasis (kala azar) is caused by two species of *Leishmania*, *L. donovani* in east Africa and south Asia and *L. infantum* in South and Central America, the Middle East and the Mediterranean basin [176]. It is spread by the bite of a sandfly and where the domestic dog can act as reservoir host [177]. The parasite migrates to the spleen and bone marrow, causing hepatosplenomegaly and bone marrow suppression. Treatment directed at reducing the visceral parasitic load frequently causes the Leishmania amastigotes to migrate back towards peripheral tissues, including the eyes, where the subsequent intense chronic inflammation causes severe tissue damage and visual loss [178]. The conjunctivae and orbits may become swollen due to infiltration with amastigotes. Post-kala azar ocular leishmaniasis is to be distinguished from primary leishmania lesions on the eyelids causing chronic localised swelling and lymphadenopathy (analogous to Romaña’s sign in Chagas disease), which can also be a feature of cutaneous and mucocutaneous forms of leishmaniasis. Treatment of visceral leishmaniasis can be very lengthy and complex, and eradication may not be achieved [179]. Importantly, therefore, these patients should not be treated with secondary immunosuppressive therapeutic agents, such as biologic anti-TNF drugs, but with repeated cycles of anti-parasitic drugs.

Helminthic parasites include roundworms (nematodes), tapeworms (cestodes) and flukes (trematodes). While some helminth infections pose significant regional public health problems, most are individually rare, yet collectively, these constitute a global challenge to vision.

Onchocerciasis is stated to be the second most common infectious cause of visual impairment after trachoma, affecting approximately three-quarters of a million people [180]. The pathogenic cause is a filarial nematode worm, *Onchocerca volvulus*, transmitted by the bite of species of blackfly near fast-flowing rivers in hyperendemic areas, most of which are in western and central Africa with smaller foci in South America and Yemen. The adult worms form nodules (onchocercomas) over bony prominences, where the female worm produces around a thousand microfilarie each day. These infiltrate the skin, eliciting inflammation that provokes intense itching. Eventually, the microfilariae infiltrate the ocular tissues, causing a spectrum of disease that includes sclerokeratitis and endophthalmitis leading to atrophic degeneration of the uveal tissues and retina, optic nerve atrophy and secondary cataract [181], although there may be regional variation in the patterns seen. Like trachoma, onchocerciasis is decreasing as a global cause of visual loss because of public health measures including vector control and mass drug administration of ivermectin, freely donated by the manufacturer [182]. The severe itching is relieved for approximately one year by annual administration of ivermectin, which greatly facilitates uptake of the mass drug administration, in turn reducing the incidence of blindness very favourably.

A particular challenge with treatment arises in areas where onchocerciasis is co-endemic with infection with *Loa loa*, another filarial worm transmitted by the deerfly in equatorial forests. Ivermectin can precipitate a fatal encephalopathy in people with high microfilarial loads of *L. loa*, whereas diethyl carbamazine, used to treat Loaiasis, can trigger the Mazzotti reaction, a life and sight threatening allergic response, in people infected with *O. volvulus* [183]. To mitigate this, the ‘test-and-not-treat’ strategy has been adopted, whereby *L. loa* microfilarial loads are quantified, and if found to be high, treatment with ivermectin deferred [184]. In such cases, albendazole has been used to decrease the *L. loa* microfilaraemia [185], while doxycycline is thought to be effective against *O. volvulus* by targeting its endogenous symbiont, *Wohlbachia* spp. [186]. *Loa loa*, the African eye worm, is known for its frequently dramatic appearance in the subconjunctival space, but it does not commonly cause visual loss except in rare cases when it invades the intraocular space [187].

Ocular toxocariasis is another nematode infection, caused by accidental ingestion of the eggs of *Toxocara* spp. shed in the faeces of cats (*T. cati*) and dogs (*T. canis*), and it can be acquired worldwide [188]. The larvae hatch in the intestine, penetrate the intestinal wall and migrate haematogenously to the eye, termed ocular larva migrans, almost always in the absence of systemic disease [189]. Ocular toxocariasis is typically a unilateral condition, tending to present as a posterior pole granuloma, as a peripheral granuloma with fibrous bands to the posterior retina, or as a chronic endophthalmitis, frequently resulting in severe visual loss [190].

The clinical finding of a motile retinal worm on fundoscopy, in association with optic disc swelling and macular exudates, was originally attributed to ocular toxocariasis [191]. Originally described as diffuse unilateral subacute neuroretinitis, it is now understood occasionally to affect both eyes [192] and is perhaps more accurately termed diffuse subacute neuroretinitis (DSN) [193]. In the early stages, there may be mild central visual loss, and disc swelling, macular exudates and clusters of small white retinal lesions may be visible on fundoscopy. These lesions may progress to profound visual loss with optic atrophy and widespread degeneration of the retinal pigment epithelium. Following the observation that this condition also could be caused by an intestinal racoon nematode, *Baylisascaris procyonis* [194], many additional nematodes have been implicated, including soil-transmitted helminths, *Strongyloides* spp., food- or waterborne zoonotic helminths, arthropod-borne filarial worms and non-human hookworms [195].

Soil-transmitted helminth infections (*Ascaris lumbricoides*, hookworms *Ancylostoma duodenale* and *Necator americanus*, and *Trichuris trichiuria*) are all passed through human faeces, and are common throughout the tropics, especially in agricultural areas where open defecation is practiced, and shoes are not always worn [196]. Mass drug administration with anti-helminthic drugs is used to limit their morbidity in some countries. Infection with *Strongyloides stercoralis*, also transmitted through soil in endemic areas, often can be latent until the immune system is suppressed (or senescent), after which it can disseminate throughout the body with occasionally fatal effects [197]. These nematodes have in common the ability to penetrate human skin and migrate through subcutaneous tissues and blood vessels, entering the right heart circulation and eventually finding their way to the ocular tissues. Similarly, canine hookworm eggs (*Ancylostoma* spp.) are passed through dog faeces, from which they develop into larvae capable of penetrating human skin, for instance on beaches frequented by both dogs and humans. Infection is normally limited to (highly pruritic) cutaneous larva migrans on exposed body parts, but aberrant migration to the eye is also possible.

Food- and waterborne zoonotic helminthic eye infections can be transmitted through copepods [198], tiny crustaceans found virtually ubiquitously in bodies of water. Gnathostomiasis, another nematode cause of DSN [199] found mostly in southeast Asia, is one such example. *Gnathostoma* spp. eggs are passed through the faeces of definitive animal hosts into bodies of water, whereupon the eggs embryonate and form larvae which are ingested by copepods as the first intermediate host. These in turn are consumed by second intermediate hosts such as frogs, fish, snakes and ducks. Humans become infected by eating the undercooked meat of these animals, or possibly by drinking contaminated water, or even by applying the flesh of infected animals as a poultice for the eye [200]. Besides causing DSN, *Gnathostoma* spp. can prove highly destructive to the eye, invading orbital tissues [201] and the intraocular compartment [202] alike. It is probably a matter of chance whether a helminth capable of migrating through tissue ends up in the retina, the anterior chamber or the orbit.

In contrast, the rat lungworm, *Angiostrongylus cantonensis*, is not transmitted through copepods but through eating undercooked land snails [203]. The snails become infected by consuming eggs present in rat faeces [204]. Outbreaks of angiostrongyliasis have been known to occur at restaurants where snails are considered a delicacy. Considered endemic in some parts of eastern Asia, *A. cantonensis* is known to have spread widely [205]. Larvae from these nematodes can invade all ocular tissues, as well as the central nervous system and the optic nerve [203, 206].

Diffuse neuroretinitis has also been attributed to filarial nematode worms transmitted by mosquitoes, such as *Brugia malayi* [207], which is endemic in tropical coastal regions in south Asia. Humans are definitive hosts for these and other filarial parasites around the tropical belt, such *Wuchereria bancrofti*, which has been known to present as panuveitis [208].

Vector-borne transmission is possible even in temperate climates, such as southern Europe. *Dirofilaria* spp. (including the dog heartworm) are endemic in many parts of the world, including the Mediterranean, the primary hosts being wild and domestic canids and felids [209]. Humans can become infected following the bite of a mosquito vector, resulting in pulmonary or subcutaneous dirofilariasis. Ectopic infections in the orbital and ocular tissues are possible with some dirofilarial species and may cause severe visual loss.

*Trichinella* spp. is a nematode parasite with a worldwide distribution, commonly infecting domestic and wild pigs, as well as other wild animals [210]. The larvae encyst in striated muscle and are liberated by gastric juices in humans eating undercooked pork, especially in temperate climes. The female adult worms can burrow into the intestinal mucosa, passing larvae directly into the tissues and circulation to disseminate widely, including to the orbit, where they can cause chronic peri-orbital oedema and damage to intraocular structures [211]. While measures to raise food standards, such as freezing meat, have greatly reduced the incidence of trichinellosis in rich countries, the consumption of wild game perpetuates the risk of human infection.

The domestic pig is the intermediate host for *Taenia solium*, one of the better-known tapeworm infections to affect the eyes [212]. Consumption of measly pork containing encysted larvae (cysticerci) results in human tapeworm infection. The adult tapeworm attaches itself to the mucosal wall of the small intestine, shedding ova. The eggs may be ingested via faeco-oral autoinfection, or via contaminated food, following which the larvae penetrate the gut wall and migrate to any site in the body to form cysticerci. Most commonly, ophthalmic manifestations are orbital, although cysts may also appear in the vitreous cavity, the subretinal space and the anterior chamber. Ova shed by the dog tapeworm, *Echinococcus granulosus*, which causes hydatid disease, may occasionally be inadvertently ingested by humans. Rarely, cysts may seed the visual pathways and ocular structures causing severe sight impairment [213].

Sparganosis is another example of copepod transmission, in this case caused by the larvae of intestinal tapeworms, *Spirometra* spp. [214], present in domesticated and wild animals. The eggs are shed in the faeces of infected animals, and embryonate in water, releasing ciliated larvae which are ingested by copepods and undergo further stages of development. The copepods are consumed by fish and amphibians, providing a route for the *Spirometra* larvae to enter the human food chain. These larvae can cross the intestinal wall and migrate to ocular or orbital tissues, eliciting severe inflammation and eventual visual loss [215].

While trematodes have been reported to cause DSN [216] the most common ocular manifestation of a systemic trematode infection is probably presumed trematode-induced granulomas or granulomatous anterior uveitis, which has been reported in various tropical and subtropical countries where swimming in fresh water containing the snail host of various flukes risks infection, including *Schistosoma* spp. [217]. and *Procerovum varium* [218]. The trematode larvae (cercariae) penetrate the skin and are carried in the circulation to distal sites throughout the body, where they mature and lay eggs. Migration to the vessels surrounding the eye may result in ectopic ova, producing a granuloma in the anterior chamber or the ciliary body. In sheep-rearing countries, flukes such as *Fasciola hepatica* may rarely reach the eye ectopically [219], possibly following consumption of watercress contaminated with larval cysts.

Rare arthropod infections causing visual loss include internal ophthalmomyiasis, caused by the larvae of a variety of flies around the world, such as the sheep bot fly *Oestrus ovis* [220]. Once settled on the conjunctival surface, these larvae can burrow into the eye, forming characteristically long and wide subretinal tracks, and sometimes they appear in the vitreous cavity. Another rare arthropod eye infection, known as pentastomiasis, can occur following ingestion of undercooked snake meat infected with *Armillifer armillatus* [221], or by accidental ingestion of a canid nasopharyngeal parasite, *Linguatula serrata*, known as tongue worm [222]. These large organisms can cause severe destruction of the ocular tissues, leading to monocular blindness.

## Summary

While no review on infectious ophthalmic disease can be comprehensive, it is a useful exercise to consider the spectrum of organisms that can infect the human eye, and the impact this group of diseases has on ocular morbidity and sight impairment. Together, these conditions cast a formidable shadow on global efforts to prevent unnecessary visual loss. Some of the major sight-threatening infections, such as herpesviruses infections, infectious keratitis and orbital cellulitis, as well as lesser-known entities like diffuse subacute neuroretinitis are often unilateral, whereas other common infections such as ocular tuberculosis and acquired toxoplasmosis are frequently asymmetric. The recognition of monocular visual impairment as a form of disability should drive more prevalence studies to evaluate the morbidity of these conditions.

As efforts to contain and suppress the major blinding infectious diseases, such as trachoma and onchocerciasis, continue to bear fruit, our attention naturally turns to other preventable eye diseases of global importance, such as cytomegalovirus retinitis, infectious keratitis and other helminthic eye infections. These, too, impose a disproportionate burden in developing societies. To capture the true burden of this unique group of diseases, it is proposed that future prevalence studies consider them in aggregate under one category. For a more comprehensive assessment, this categorisation could include the secondary sight threatening effects of infection and treatment, as well as post-infectious ocular conditions. This approach would help to direct resources, it is hoped, not just to cataract and refractive services, but also to other avoidable causes of blindness like ocular infections and inflammation which still need serious attention in many parts of the world [13, 223].

Success in halting the spread of trachoma and onchocerciasis has proven beyond doubt that collaborative multidisciplinary approaches can turn the tide against complex infectious diseases. Key interventions, of course, include vaccination, which is available for numerous viruses [224], bacteria [225] and even parasites [226]. The unprecedented success of messenger RNA vaccines in combating the SARS-CoV-2 pandemic gives much room for hope [227]. Public health initiatives to mitigate the impact of neglected tropical diseases will also reduce their visual burden, including mass drug administration [228], improvements in environmental hygiene [229], vector control [230] and improved access to healthcare [231].

The advent of telemedicine is now well-established, and many ophthalmologists count themselves among the converted, galvanised perhaps by the SARS-CoV-2 pandemic [232]. The highly visual specialty of ophthalmology, abetted by highly informative imaging, lends itself well to remote medicine, and it is envisaged to expand further as technology spreads. The widespread availability of mobile phones, enabling photography of external and, to a lesser extent, internal ocular structures [233], as well as relatively low-cost handheld retinal cameras [234], augurs well for the future of teleophthalmology. It has unmatched potential for the delivery of care in resource-poor settings, though significant challenges, including cost, electricity supply and acceptance, remain [235]. While the role of handheld fundus cameras in screening of diabetic retinopathy appears to be well established, their value in diagnosis of significantly more complex infectious eye disease presentations is as yet unclear.

We stand before a new era, as attested by apparent climate emergencies across the globe [236]. Extreme weather, degradation of the natural world, overpopulation, human incursion into animal habitats, conflict, international travel and food poverty conspire to stimulate the proliferation of vector-borne, zoonotic and other infectious diseases, while stymying human efforts to contain them, with untold consequences [231, 237]. What this portends for the future of human health and health systems remains a subject of intense debate, but it seems likely that infectious diseases, including those that threaten vision, will take centre stage [238]. Community-based surveillance programmes to detect and respond to outbreaks are already operational [239], and their scope is set to expand. In addition, WHO has recently launched a global network to detect infectious disease threats, harnessing the power of pathogen genomics across the globe [240]. At the same time, One Health Networks have emerged, seeking to address health inequality at a global level through multisectoral, transdisciplinary and community-orientated collaboration [241]. There is no question that ophthalmologists too will have a part to play, for example in reporting and managing presentations compatible with Emerging Infectious Diseases [11]. Global reporting networks may ultimately help to lift the veil on the true global burden of infectious eye disease.

Ultimately, sophisticated artificial intelligence (AI) systems may be required to process the intricate interplay between incoming information on diagnosis and epidemiological mapping of infectious eye disease on one hand and the mobilisation of resources and collaborative public health initiatives on the other, simultaneously factoring in ever-increasing environmental threats to healthcare [242]. Already established in various domains of ophthalmic healthcare [243], AI appears set to open the doors to a new era of information gathering and synthesis, transforming ophthalmology in hitherto unimagined ways.
